# Prevalence and risk factors of pressure ulcer in hospitalized adult patients; a single center study from Ethiopia

**DOI:** 10.1186/s13104-018-3948-7

**Published:** 2018-11-29

**Authors:** Dinkie Tadele Bereded, Mohammed Hassen Salih, Abebaw Eredie Abebe

**Affiliations:** 1 Dessie Health Science College, Dessie, Ethiopia; 2 School of Nursing, College of Medicine & Health Sciences, University of Gondar, Gondar, Ethiopia

**Keywords:** Pressure ulcer, Prevalence, Braden scale, Patient, Cross-sectional

## Abstract

**Objective:**

The main objective of this study was to assess the prevalence of pressure ulcer and its risk factors among adult hospitalized patients at Dessie Referral Hospital, Northeast Ethiopia, 2016 G.C

**Result:**

A cross-sectional institutional based study with a single population proportion formula was used to determine the sample size. The total sample size of 355 patients was distributed proportionally to the respected wards. Every other patient was selected by systematic random sampling technique from each ward with a response rate of 100% A total of 53 patients with pressure ulcer were detected giving the prevalence rate of 14.9%. The lack of regular positioning and activity, friction/shear, and prolonged hospitalization were risk factors for pressure ulcer.

## Introduction

Pressure ulcer (PU) also called bed sore is an area of localized damage to the skin and underlying tissue caused by pressure, shear, friction or a combination of these [1, 2]. PU ranges from areas of slightly discolored skin to area of deep purulent wound cavities that extend to underlying muscle and bone [3]. The amount of time it takes for PU to develop varies from as short as 2 h in patients at greatest risk. PU is a major cause of morbidity and mortality and one of the important measures of the quality of clinical care in a healthcare setting [4–6].

PU remains one of the major health problems around the world. For every 1,000,000 patients who developed PU 65,000 die from complications which presents a major health challenge worldwide [7, 8]. And also an 80% increase in the number of patients who were hospitalized from the year 1993 to 2006, which leads to PU occurrence [8].

As studies revealed, PU is common in high and middle-income countries but it is rarely researched in low-income countries A prevalence of 12.7% was reported in Brazil [9], 10.4% in Turkey [10], a higher prevalence of 47.6% in Thailand [11], and 16% in Ethiopia [12]. Also, studies on health professional knowledge about pressure ulcer in Uganda and Ethiopia [13, 14].

Moreover, researchers identified variety of factors that influence the occurrence of PU including friction/shear, moisture sensory perception, immobility, the position of the patient, length of hospital stay (LHS), gender, nutritional status of the patient, age and use of medical relieving devices [12, 15–18].

Therefore, assessing the prevalence and identifying the risk factors that influence the occurrence of PUs in resource-limited countries is important for possible interventions in the prevention of PU and increases the quality of care.

## Main text

### Methods

Institution-based cross-sectional study was conducted in Dessie Referral Hospital, Northeast Ethiopia, from March to April 2016. This hospital has different wards. Among these Medical, Surgical, Gynecological, and Orthopedic wards were selected for this study.

All adult admitted patients and avail during the study period were the source and study population respectively. Patients who didn’t develop PU prior to admission and who stayed in the hospital ≥ 24 h were included but those patients who are critically ill were excluded from the study.

PU was the outcome) variable and sociodemographic variables, Braden risk assessment tool, clinical characteristics (like presence and type of chronic illness) and presence of medical relieving device were used for the independent variable.

The sample size was determined by using a single population proportion formula considering the following assumptions: prevalence (p) of pressure ulcer 16% taken from a recent study conducted in Ethiopia [12]. Z = standard normal distribution value at 95% confidence level of Z^a/2^ = 1.96, and margin of error (*w*) = 5%. This gave a sample size of 323 admitted patients. Considering 10% nonresponse rate, the total sample size was 355.

Systematic random sampling technique was used to select every other patient and the first patient was selected by using lottery method.

Structured questionnaires which were developed by searching different scholars and a previous research paper on a similar study. And also physical examination to assess the presence or absence of PU and risk assessment by using checklist adapted from the Braden scale was used to collect data [8, 9].

The questionnaire was transcribed into Amharic (local language) by language experts and translated back to English by another language expert for consistency. Also, physical examination performed to assess and grade PU. Four B.Sc. nurses were used to collect data and two-second degree nurses assigned for supervision. Five-day training was given for both data collectors and supervisors. Data entry was done by using EPI Info version 7 statistical software and exported to SPSS version 20.0 software package for analysis.

Data quality was controlled by pre-testing the questionnaire on 36 admitted adult patients in another hospital (Borumeda) with the same setting. The presence of the association between outcome and independent variables was assessed using odds ratio (OR) with 95% confidence interval by binary logistic regression model. Those variables having *p* value ≤ 0.2 in binary logistic regression model were entered into the multivariate analysis using forward ward likelihood ratio method. The final result was interpreted based upon their association of significance i.e. p ≤ 0.05.

### Results

#### Socio-demographic and clinical characteristics of the patient

A total of 355 admitted patients with a response rate of 100% were included in this study. More than half of them were rural residents. The majority (52.1%) of the respondents were females. The mean (± standard deviation) age of the respondents was 37.2 (± 13.7) and the majority (43.7%) of them fall in the age range of 33–54. Most of the patients (65.1%) were married. About 31% of the patients were unable to read and write. 28.2% of them had a chronic illness and from this, 32.4% were diabetic (Table 1).

**Table 1:** Socio-demographic and clinical characteristics of adult patients who were admitted at DRH, Northeast Ethiopia, 2016 G.C (N = 355)

Variables	Frequency	Percent
Age (in years)		
18–32	146	41.2
33–54	156	43.9
> 54	53	14.9
Sex		
Male	170	47.9
Female	185	52.1
Place of residence		
Urban	167	47
Rural	188	53
Marital status		
Single	73	20.6
Married	231	65.1
Divorced	32	9
Widowed	19	5.4
Educational status		
Illiterate	110	31
Read and write	56	15.8
1–4 grade	34	9.6
5–8 grade	56	15.8
9–10 grade	58	16.3
> 10 grade	41	11.3
Presence of chronic illness		
Yes	100	28.2
No	255	71.8
Types of chronic disease		
Diabetes mellitus	33	32.4
Cardio vascular disease	25	24.5
Respiratory disease	12	11.8
Hypertension	14	13.7
Others	18	17.6
Presence of pressure ulcer		
Yes	53	17.5
No	302	82.5
Stage of pressure ulcer		
Stage 1	34	64
Stage 2	16	30
Stage 3	3	6
Anatomical site		
Sacral	26	49.1
Heel	12	22.6
Elbow	4	7.5
Occiput	2	3.8
Shoulder	2	3.8
Sacral and shoulder	3	5.7
More than two sites	4	7.5

*DRH* Dessie Referral Hospital, *GC* Gregorian calender

#### Prevalence and stages of pressure ulcer

A total of 53 PU was detected, with the prevalence rate of 14.9% (11.2–18.9). The prevalence was higher among male respondents and the sacral anatomical site was the main one. Based on the European Pressure Ulcer Advisory Panel (EPUAP) grading scale 34 (64%) developed stage I PU (Table 1).

#### Braden scale pressure ulcer risk assessment characteristics of adult patients

Hundred sixty-six (46.8%) of the respondents had no impairment in their sensory perception and 168 (47.3%) walked frequently in their activity. Regarding mobility, 151 (42.2%) of patients had no limitation. Of the total admitted patients 205 (57.7%) were rarely moist, two-thirds (60.6%) of them had no apparent problem in their friction/shear and one-third (31.3%) of them had probably inadequate nutrition (Table 2).

**Table 2:** Braden scale pressure ulcer risk assessment characteristics of respondents who were admitted at DRH, Northeast Ethiopia, 2016 G.C (N = 355)

Variables	Frequency	Percent	Remark
Sensory perception			
Completely limited	74	20.8	
Very limited	61	17.2	
Slightly limited	54	15.2	
No impairment	166	46.8	
Moisture			
Constantly moist	48	13.5	
Very moist	38	10.7	
Occasionally moist	64	18	
Rarely moist	205	57.7	
Activity			
Bedfast	60	16.9	
Chair fast	40	11.3	
Walks occasionally	87	24.5	
Walks frequently	168	47.3	
Mobility			
Completely immobile	66	18.6	
Very limited	42	11.8	
Slightly limited	96	27	
No limitation	151	42.2	
Nutrition			
Very poor	59	16.6	
Probably inadequate	111	31.3	
Adequate	142	40	
Excellent	43	12.1	
Friction and shear			
Problem	59	16.6	
Potential problem	81	22.8	
No apparent problem	215	60.6	

*DRH* Dessie Referral Hospital, *GC* Gregorian calendar

#### Factors associated with the occurrence of pressure ulcer

All independent variables were analyzed in binary logistic regression with the dependent variable to know their association. Among those variable LHS, the presence of chronic illness, use of medical devices, use of pressure relieving devices, position change, sensory perception, moisture, activity, mobility, friction/shear were found to be significant in binary logistic regression and then taken into multivariate analysis.

Those patients whose LHS was 7–20 days were 8.44 times more likely to develop PU than patients who were stayed for ≤ 6 days, patients who had chair and bedfast in their activities were found to be 11 times more likely to develop PU than those patients who were walked frequently, patients who had a problem of friction/shear had 16.4 times more risk to develop PU than those who had no apparent problem in friction/shear and patient’s position change also the other independent variable which was found to be associated with the PU. Those patients who were not changed their position had 10.42 times higher risk to develop PU than those who changed their position every 2–3 h.

### Discussion

The prevalence of PU in this study was in line with studies conducted in Ethiopia (16%) [12], Germany 11.7% [19], Brazil (12.7%) [9], Turkey (11.7%) [10]. However, higher than international survey [20]. The possible explanation for this difference (higher prevalence rate) might be due to lack of advanced nursing facility and materials in the hospital, absence of risk assessment tool, lack of guideline for prevention and treatment of pressure ulcer which leads to poorly assessing the risks.

The prevalence is lower than the study conducted in Sweden (22.9%), Italy (27%), Australia (22%) and Thailand (47.6%) [11, 21–23].

LHS in this study significantly associated with PU. Patients whose LHS was 7–20 days were 8.44 times more likely to develop PU than those patients who were stayed for ≤ 06 days. This finding is consistent with studies conducted in Ethiopia and Sweden [12, 24]. When patients LHS increases, the risk of hospital-acquired infection increases which leads to the development of PU.

Activity is significantly associated with PU. Patients who had activity restriction (to chair and bed) were 11 times and 7.58 times respectively more likely to develop PU than those patients who walked frequently. This result is in line with the study conducted by Australian medical association [25]. The possible explanation for this might be due to the prolonged pressure on bony prominence which interferes circulation to the underlying skin and which in turn decreases the skin resistance to pressure (Table 3).

**Table 3:** Association between some selected variables and pressure ulcer at DRH, Northeast Ethiopia, 2016 G.C (N = 355)

Variable	Pressure ulcer		COR (95% CI)	AOR (95% CI)	Remark
	No	Yes			
Length of stay (days)					
≤ 6	145 (96.7%)	5 (3.3%)	1	*1*	
7–20	143 (82.7%)	30 (17.3%)	6.1 (2.3, 16)*	8.44 (2.1, 34)**	
≥ 21	8 (33.3%)	16 (66.7%)	58 (16.9, 198)*	7 (10, 52)**	
Pressure relieving device					
Yes	49 (96.1%)	2 (3.9%)	1		
No	253 (83.2%)	51 (16.8%)	4.94 (1.16, 20)		
Use of medical device					
Yes	78 (75.5%)	25 (24.3%)	2.6 (1.4, 4.66)		
No	229 (88.9%)	28 (11.1%)	1		
Chronic illness					
Yes	72 (72%)	28 (28%)	3.58 (1.4, 4.66)*	2.66 (0.78, 9)	
No	230 (90.2%)	25 (9.8%)	1		
Change position					
Yes	203 (97.1%)	6 (2.9%)	1	*1*	
No	99 (67.8%)	47 (32.2%)	16 (6.64, 38.8)*	10.42 (2.9, 37)**	
Sensory perception					
Completely limited	49 (66.2%)	25 (33.8%)	27.7 (8, 95.7)	1.85 (0.35, 9.8)	
Very limited	40 (65.6%)	21 (34.4%)	28.5 (8, 100)	1.57 (0.24, 10.4)	
Slightly limited	50 (92.6%)	4 (7.4%)	4.4 (0.94, 20.8)	0.63 (0.47, 8.25)	
No impairment	163 (98.2%)	3 (1.8%)	1	1	
Moisture					
Constantly moist	25 (52.2%)	23 (47.8%)	22.6 (9.16, 56)	2.81 (0.55, 15)	
Very moist	21(56.8%)	17 (44.7%)	19.9 (7.7, 51.7)	4.5 (0.82, 25).	
Occasionally moist	59 (96.1%)	5 (7.8%)	2.12 (0.66, 6.6)	0.7 (0.09, 5.3)	
Rarely moist	197 (95.6%)	8 (3.9%)	1	1	
Activity					
Bed fast	28 (46.7%)	32 (53.3%)	26.28 (10.6, 65)*	*7.58 (1.7*, 32)**	
Chair fast	28 (70%)	12 (30%)	9.86 (3.6, 27.2)*	*11 (2*, 61)**	
Walks occasionally	85 (97.7%)	2 (2.3%)	0.54 (0.11, 2.66)*	*0.42 (0.043, 4.1)*	
Walks frequently	161 (995.6%)	7 (4.2%)	1	*1*	
Friction/shear					
Problem	17 (28.8%)	42 (7127%	86.06 (32, 231)	16.4 (4.4, 61)**	
Potential problem	76 (93.8%)	5 (6.2%)	2.29 (0.68, 7.7)	*11 (0.2, 5.6)*	
No apparent problem	209 (97.2%)	6 (2.8%)	1	*1*	

The main objective of this study was to assess the prevalence of pressure ulcer and its risk factors among adult hospitalized patients at Dessie Referral Hospital Northeast Ethiopia, 2016 G.C Hospital

Strongly associated variables are in italic

*COR* crude odd ratio, *AOR* adjusted odd ratio, *DRH* Dessie Referral, *GC* Gregorian calendar

** p-value ≤ 0.05, * p-value ≤ 0.20 in binary logistic regression

The odds of developing Pu among patients who had a problem of friction/shear was 16.4 times more likely than those who had no apparent problem in friction/shear. This finding is in line with the study conducted In Northwest Ethiopia and Thailand [11, 12].

Patient’s position change was also the other independent variable which was significantly associated with PU. Those patients who did not have their position changed were 10.42 times more likely to develop PU than those who had their position changed every 2–3 h. This finding was comparable with the result by an International review of PU prevention [26].

### Conclusion and recommendation

The prevalence of PU was high. LHS, activity, friction/shear, and changed position were significantly associated. We recommend for the Ministry of Health to establish a better system to early identify risk factors towards the occurrence of PU and implement different tools like Braden scale pressure ulcer risk assessment tool and prepare guideline on how to handle patients on PU. For healthcare provider’s contribution is vital in the prevention of PU for admitted patients. Therefore, it would better to strive for standards of care in the health care system. Also, for researchers conducting a prospective study to examine the incidence and associated factors of PU for hospitalized patients.

## Limitation

Firstly, the cross-sectional design of this study limits our ability to make causal inferences. Second, time and resource limitation for not doing a prospective study. Thirdly, a patient who already developed a PU was not grouped on their disease type.

## Abbreviations

AOR: adjusted odds ratio
COR: crude odd ratio
DRH: Dessie Referral Hospital
EPUAP: European Pressure Ulcer Advisory Panel
GC: Gregorian calendar
HAPU: hospital-acquired pressure ulcers
LHS: length of hospital stay
MW: medical ward
NPUAP: National Pressure Ulcer Advisory Panel
OR: odds ratio
PU: pressure ulcer
SW: surgical ward

## References

[1] Ceelen K. Aetiology of pressure ulcers A literature review. Part I of MSc-thesis, Eindhoven university of Technology, Faculty of Biomedical Engineering. 2003.

[2] Keller BPJA, Wille J, van Ramshorst B, Wvd C (2002). Pressure ulcers in intensive care patients: a review of risks and prevention. Intensive Care Med.

[3] Panel EPUA. National Pressure Ulcer Advisory Panel. Prevention and treatment of pressure ulcers: guick reference guide Washington DC National Pressure Ulcer Advisory Panel; 2009.

[4] Benbow M (2006). Guidelines for the prevention and treatment of pressure ulcers. Nurs Stand (through 2013).

[5] Berlowitz D, Jini J (2014). Incidence and prevalence of pressure ulcers. Pressure ulcers in the aging population.

[6] Bluestein D, Javaheri A. Pressure ulcers: prevention, evaluation, and management. Am Fam Physician. 2008;78(10). 19035067

[7] Macgregor L (2009). International guidelines. Pressure ulcer prevention: prevalence and incidence in context. A consensus document.

[8] Soban LM, Hempel S, Munjas BA, Miles J, Rubenstein LV (2011). Preventing pressure ulcers in hospitals: a systematic review of nurse-focused quality improvement interventions. Jt Comm J Qual Patient Saf.

[9] Chacon JMF, Blanes L, Hochman B, Ferreira LM (2009). Prevalence of pressure ulcers among the elderly living in long-stay institutions in São Paulo. Sao Paulo Med J.

[10] Inan DG, Öztunç G (2012). Pressure ulcer prevalence in Turkey: a sample from a university hospital. J Wound Ostomy Continence Nurs.

[11] Suttipong C, Sindhu S (2012). Predicting factors of pressure ulcers in older Thai stroke patients living in urban communities. J Clin Nurs.

[12] Gedamu H, Hailu M, Amano A (2014). Prevalence and associated factors of pressure ulcer among hospitalized patients at Felegehiwot referral hospital, Bahir Dar, Ethiopia. Adv Nurs.

[13] Mwebaza I, Katende G, Groves S, Nankumbi J (2014). Nurses’ knowledge, practices, and barriers in care of patients with pressure ulcers in a Ugandan teaching hospital. Nurs Res Pract.

[14] Dilie A, Mengistu D (2015). Assessment of nurses’ knowledge, attitude, and perceived barriers to expressed pressure ulcer prevention practice in Addis Ababa government hospitals, Addis Ababa, Ethiopia, 2015. Adv Nurs.

[15] Nuru N, Zewdu F, Amsalu S, Mehretie Y (2015). Knowledge and practice of nurses towards prevention of pressure ulcer and associated factors in Gondar University Hospital, Northwest Ethiopia. BMC Nurs.

[16] Saunders LL, Krause JS, Peters BA, Karla S, Reed M (2016). The relationship of pressure ulcers, race, and socioeconomic conditions after spinal cord injury. J Spinal Cord Med.

[17] Defloor T (1999). The risk of pressure sores: a conceptual scheme. J Clin Nurs Res Pract.

[18] Bergstrom N (1987). The Braden Scale for predicting pressure sore risk. Nurs Res.

[19] Lahmann NA, Halfens RJ, Dassen T (2005). Prevalence of pressure ulcers in Germany. J Clin Nurs.

[20] Catherine VanGilder M, Amlung S, Harrison P, Meyer S (2009). Results of the 2008–2009 International Pressure Ulcer Prevalence™ Survey and a 3-year, acute care, unit-specific analysis. Ostomy Wound Manag.

[21] Gunningberg L, Stotts NA (2008). Tracking quality over time: what do pressure ulcer data show?. Int J Qual Health Care.

[22] Capon A, Pavoni N, Mastromattei A, Di Lallo D (2007). Pressure ulcer risk in long-term units: prevalence and associated factors. J Adv Nurs.

[23] Prentice JL, Stacey M, Lewin G (2003). An Australian model for conducting pressure ulcer prevalence surveys. Prim Intent: Aust J Wound Manag.

[24] Gunningberg L, Stotts NA, Idvall E (2011). Hospital-acquired pressure ulcers in two Swedish County Councils: cross-sectional data as the foundation for future quality improvement. Int Wound J.

[25] Woodward M (1999). Risk factors for pressure ulcers-can they withstand the pressure?. Prim Intent.

[26] International review. Pressure ulcer prevention: pressure, shear, friction and microclimate in context. A consensus document. London; 2010.

